# Genetic characterization of *Contracaecum* cf. *overstreeti* (Nematoda: Anisakidae) larvae in *Mugil cephalus* fish from the pacific coast of Ecuador

**DOI:** 10.1016/j.ijppaw.2025.101185

**Published:** 2025-12-31

**Authors:** Manuel Calvopina, Carlos Bastidas-Caldes, Fernanda Hernández-Alomía, William Cevallos, Richar Rodríguez-Hidalgo, Hiromu Sugiyama

**Affiliations:** a One Health Research Group, Facultad de Medicina, Universidad De Las Américas, (UDLA), Quito, 170124, Ecuador; b School of Medicine, Universidad de Especialidades Espíritu Santo, Samborondón, Ecuador; c Dirección General de Investigación, Universidad De Las Américas, 170125, Quito, Ecuador; d Instituto Nacional de Biodiversidad (INABIO), Quito, Ecuador; e Facultad de Medicina, Universidad Central Del Ecuador, Quito, 170521, Ecuador; f Instituto de Investigación en Zoonosis, Universidad Central Del Ecuador, Quito, 170521, Ecuador; g Facultad de Medicina Veterinaria y Zootecnia, Universidad Central Del Ecuador, Quito, 170521, Ecuador; h Department of Parasitology, National Institute of Infectious Diseases, Tokyo, Japan

**Keywords:** *Contracaecum overstreeti*, Anisakidae, *Mugil cephalus*, Fish, Food safety, Ecuador

## Abstract

This study reports the genetic characterization of Anisakidae larvae infecting the flathead grey mullet (*Mugil cephalus*) fish. Larvae were recovered from fish captured by artisanal fisheries and sold in a coastal town in northwestern Ecuador. In total, 19 larvae were obtained from nine fish, all larvae were exclusively encysted within the muscle tissue. Molecular identification was performed by PCR amplification and DNA sequencing of the nuclear ribosomal region ITS1-5.8 S-ITS2, the mitochondrial cox2 gene, and the EF-1α gene. Comparative analyses with sequences available in GenBank, followed by phylogenetic reconstruction, confirmed the larvae as *Contracaecum overstreeti*. This constitutes the first molecular evidence of *C. overstreeti* in this edible fish from Ecuador and provides new host–parasite records. The identification of this zoonotic nematode in marine fish of human consumption underscores the need for expanded surveillance in other neotropical marine ecosystems and in other commercially important fish species and highlights potential infection to humans.

## Highlights

• In Ecuador, limited information is available on specific genetic-molecular variants of *Anisakidae* family.
• *Contracaecum overstreeti* was identified directly from sea *Mugil cephalus* fish.
• Herein we reported the potentially zoonotic *Contracaecum* spp. found in the muscle of the “lisa”, a fish often eaten raw in Ecuador.

## Graphical abstract

**Figure:**
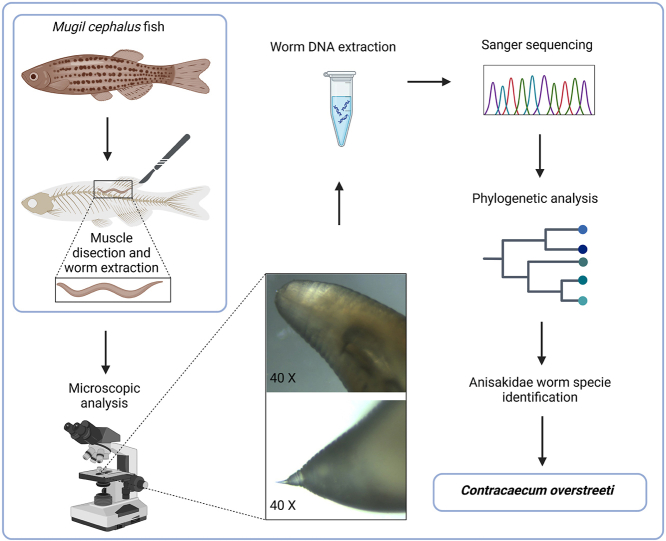

## 1 Introduction

The roundworm parasites of the Anisakidae family (Phylum: Nematoda, superfamily: Ascaridoidea) are globally distributed and recognized as an emerging fish borne parasitic disease (Acha and Szyfres, 2003). The family includes four major genera: *Anisakis*, *Pseudoterranova*, *Phocanema*, and *Contracaecum*. These four groups show high genetic diversity, suggesting the presence of cryptic species (Bao et al., 2023; CDC, 2019; Nagasawa, 2012). Anisakidae include members of 1) the *Anisakis simplex* complex (*A. simplex* sensu stricto, *A. pegreffii*, *A. berlandi*), other species is included as Skrjabinisakis spp. (formerly *Anisakis paggiae*, *brevispiculata* and *physeteris*); 2) the genus *Pseudoterranova* harbours two species infecting kogiid whales *Ps. Kogiae* and *Ps. Ceticola*, 3) the genus *Phocanema*, with *Ph. decipiens* (sensu stricto), *Ph. decipiens*, *Ph. Azarasi*, *Ph. Bulbosa*, *Ph. Cattani* and Ph. *Krabbei*, all parasites of pinnipeds, and 3) the *Contracaecum osculatum* complex (*C. osculatum* sensu stricto, A, B, D, and E or *C. overstreeti*, *C. aff. Multipapillatum*, *C. rudolphii*, and *C. gibsoni*). *Anisakis, Pseudoterranova*, and *Contracaecum* have been implicated in human infections (Chai et al., 2005; Shamsi and Butcher, 2011; Weitzel et al., 2015; Falla-Zuñiga et al., 2021).

Each genus has distinct and overlapping geographical distributions. The genus *Anisakis* occurs in both deep sea and coastal environments of the Pacific Ocean, Atlantic basin, and Alaskan coast, while those of *Pseudoterranova*, *Phocanema*, and *Contracaecum* are predominantly found in cold-water coastal environments (Bao et al., 2023; CDC, 2019). In South America, anisakid infections in edible fish are significant, with high prevalence rates reported in both Atlantic and Pacific waters (Falla-Zuñiga et al., 2021). In Venezuela, for instance, *Contracaecum* spp. was found parasitizing 100 % of Mugilidae fish (Bracho-Espinoza, 2023).

Species identification of anisakid larvae in fish hosts is challenging using only morphological methods (Chai et al., 2005; Shamsi and Suthar, 2016; Shamsi et al., 2018). Scanning electron microscopy can provide detailed morphological information (Tunya et al., 2020). Molecular genetic methods, particularly PCR and DNA sequencing of ITS rRNA and mtDNA *cox2* genes, have become the standard for reliable species identification (Zhu et al., 2002; Weitzel et al., 2015; Tunya et al., 2020; Nadler and Hudspeth, 2000; Cipriani et al., 2022).

In Ecuador, the grey mullet (*Mugil cephalus*), locally known as “lisa”, is extensively harvested through artisanal fishing and serves as a crucial protein source for the population. The country's annual per capita fish consumption is approximately 8 kg, contributing to 7 % of total animal protein intake (FAO, 2019). Traditional consumption patterns may pose health risks. “Lisa” is commonly prepared raw in “ceviche” —a dish prepared with raw fish, shrimp, or shellfish marinated in lime juice, and in “fanesca” —prepared with dried salted fish, served during Holy Week celebrations. These preparation methods can lead to human anisakidosis, characterized by gastrointestinal disturbances and/or allergic reactions (Acha and Szyfres, 2003; Chai et al., 2005; Falla-Zuñiga et al., 2021).

Despite the potential public health implications, occurrence, prevalence and molecular analyses of anisakid nematodes in Ecuadorian marine fishes remain limited. A single study has identified *Anisakis pegreffii* and *A. physeteris* (moved to genus *Skrjabinisakis*) in various commercial fish species (Castellanos et al., 2018), but there are no reports of *Contracaecum* spp. or investigations of anisakids in grey mullet. The present study reports the presence of the Anisakidae *Contracaecum overstreeti* in grey mullet fish from Ecuador's Pacific coast using molecular approaches.

## 2 Materials and methods

### 2.1 Fish specimens and site of study

**Fig. 1:**
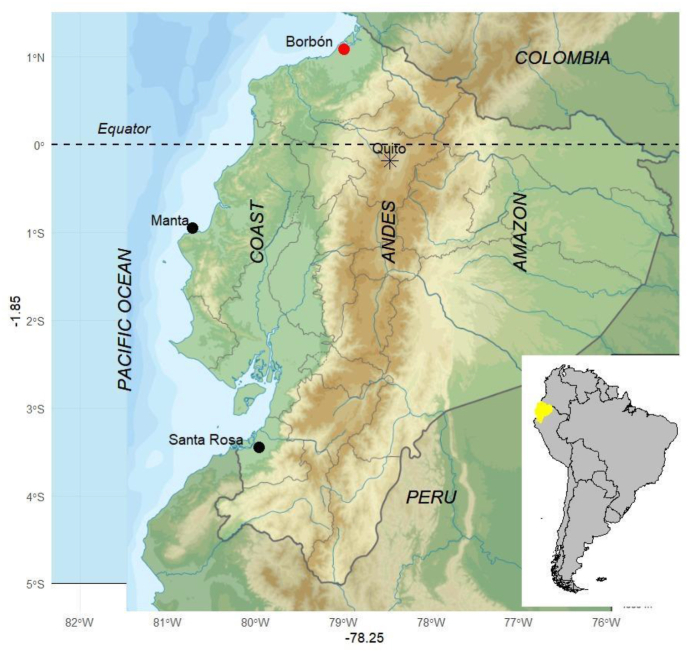
Map of Ecuador highlighting study locations. Ecuador is in northwestern South America, bordered by Colombia on the north, Peru on the east and south, and the Pacific Ocean on the west. The red dot marks Borbón town, where the 10 “lisas” fish were purchased for this study. Black dots indicate the cities of Manta and Santa Rosa, where a previous study reported the presence of *Anisakis* spp. larvae in other fish species (Castellanos et al., 2018).

We examined 10 non-eviscerated flathead grey mullet (*Mugil cephalus*) fish purchased in July 2023 from a vendor in Borbón, a coastal town on the Pacific Ocean of Ecuador (latitude 1.089202, longitude −78.98996) (Fig. 1). Borbón hosts an open marketplace where various edible fish species, caught by artisanal fishing in the Pacific Ocean, are sold.

### 2.2 Parasitological examination and identification of anisakidae

Every fish was dissected; the body cavity was opened ventrally along the middle line for visual inspection of free larvae present at the abdominal cavity. Internal organs, i.e., liver, stomach, gonads, kidney, and intestine, were also searched for larvae. After, muscle tissue was examined using non-traumatic forceps. All larvae were collected and placed into Petri dishes containing saline solution. Larvae were rinsed with distilled water and transferred into 2-mL plastic tubes, preserved in 70 % ethanol until further DNA analysis. Fresh larvae were examined using light microscopy and photographed with a digital camera (Olympus, Japan). Based on morphological features, the size, colour, presence of a boring tooth in the anterior end, the mucron in the posterior end, the opening of the excretory pore, and the characteristics of the digestive system, were pre-identified as Anisakidae family (Chai JY et al., 1986; Nagasawa, 2012; Measures, 2014).

### 2.3 Molecular and phylogenetic analyses

Using the entire anisakid larvae, DNA was extracted using PureLink™ Genomic DNA Mini Kit (Invitrogen, Carlsbad, CA, USA) according to the manufacturer's instructions. DNA was eluted with a 30 μl AE buffer and stored at −20 °C for further use. The concentration and purity of the DNA were determined by means of a Thermo Scientific™ NanoDrop™ (Thermo Fisher Scientific, Waltham, MA, USA).

Polymerase Chain Reaction (PCR) was performed targeting: (1) the 908 bp region of the ITS1-5.8 S-ITS2 with the NC5 (TAGGTGAACCTGCGGAAGGATCATT) and NC2 (TTAGTTTCTTTTCCTCCGCT) primers, PCR conditions following Zhu et al. (2002); (2) a fragment of 629 bp of the mitochondrial cytochrome oxidase subunit 2 (mtDNA *cox2*) with 211 F (TTTTCTAGTTATATAGATTGRTTTYAT) and 210 R (CACCAACTCTTAAAATTATC) primers (Nadler and Hudspeth, 2000).); and, (3) a 714 bp region of the elongation factor EF-1 alpha with *Anisakis simplex* specific primers EF-F (TCCTCAAGCGTTGTTATCTGTT) and EF-R (AGTTTTGCCACTAGCGGTTCC) according to Mattiucci et al. (2016). DNA amplification was performed in reactions of 15 μL containing 1X GoTaq® Green Master Mix (Promega, Madison, WI, USA), 0.2–0.45 μM of each primer, and 1 μL DNA. A PCR reaction without DNA sample was used as negative control. For the amplification of EF-1 alpha and ITS region, the conditions stated by Mattiucci et al. (2016) were used. For the ITS region an annealing temperature of 57 °C was performed. The PCR conditions for mtDNA *cox2* were followed as detailed by Mattiucci et al. (2014) with a slight modification for the annealing temperature at 32 °C.

PCR products were sequenced using the Sanger method on an ABI 3500xL Genetic Analyzer (Applied Biosystems, Foster City, CA, USA) with BigDye Terminator v3.1 chemistry and a capillary electrophoresis matrix. Prior to sequencing, PCR products were enzymatically purified using Exo I and FastAP. Raw sequences were edited and assembled using Molecular Evolutionary Genetics Analysis version X (MEGA X) software (Kumar et al., 2018) and subsequently compared against the GenBank database of the National Center for Biotechnology Information (NCBI) using the Basic Local Alignment Search Tool (BLAST).

The resulting sequences were deposited in the NCBI nucleotide database under the following accession numbers: PP737161, PX662930, PX662931, and PX662932 for the ITS1–5.8 S–ITS2 region, and PP740830, PX682249, PX682250, and PX682251 for the mitochondrial *cox2* gene. Sequence alignments were performed using the MUSCLE algorithm. Phylogenetic trees for both the ITS region and *cox2* gene were constructed using the neighbour-joining method under the Tamura–Nei distance model with 1000 bootstrap replicates. *Sulcascaris sulcata* sequences were included as the outgroup.

## 3 Results

**Fig. 2:**
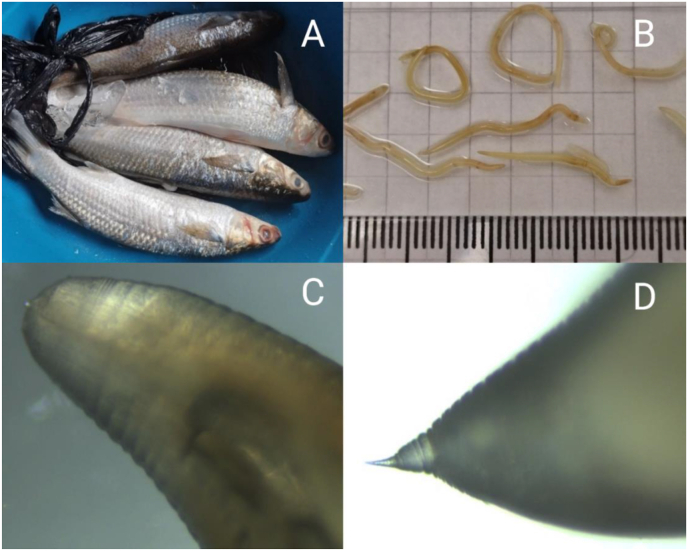
A: Four of the 10 *M. cephalus* “lisa” fish examined in this study. **B**: Entire anisakid larvae, visible to the naked eye. C: Anterior end of the larva, showing the ventral boring tooth, the cuticle shown transverse striae (40x). **D:** Posterior end of the larva, displaying the terminal pointed mucron (40x).

A total of 19 Anisakidae-type larvae were recovered from nine of the 10 *Mugil cephalus* fish (Fig. 2A). All larvae were morphologically identical (Fig. 2B). No larva was found in the abdominal cavity nor from the viscera. All larvae were isolated from the muscle tissue alongside the spinal column, predominantly in the middle.

Under light microscopy, the larvae observed were cylindrical, pale, and thick, with the body attaining its maximum width at mid-length. The median body length of the 19 specimens was 26 mm (range: 20–31 mm) (Fig. 2B). The cuticle was smooth, showing transverse striations. The anterior end exhibited a projecting boring tooth and lips (Fig. 2C). The excretory pore opened ventrally, immediately posterior of the boring tooth. The oesophagus consisted of a muscular anterior portion (proventriculus) composed of cylindrical striated fibers, measuring 1.36–1.80 mm in length (mean 1.57 ± 0.19 mm) and 0.08–0.11 mm in width (mean 0.10 ± 0.02 mm). Oesophagus was followed by a ventriculus, 0.54–0.95 mm long (mean 0.66 ± 0.14 mm) and 0.10–0.20 mm wide (mean 0.15 ± 0.04 mm), with an oblique esophago–intestinal junction. The tail was elongated, ending to a sharp mucron (Fig. 2D).

All analysed larvae yielded positive PCR amplification for both the ITS1-5.8 S-ITS2 (908 bp) and mitochondrial *cox2* (629 bp) regions. No amplification was observed for species-specific primers of *Anisakis simplex* elongation factor 1-alpha (EF-1).

**Fig. 3:**
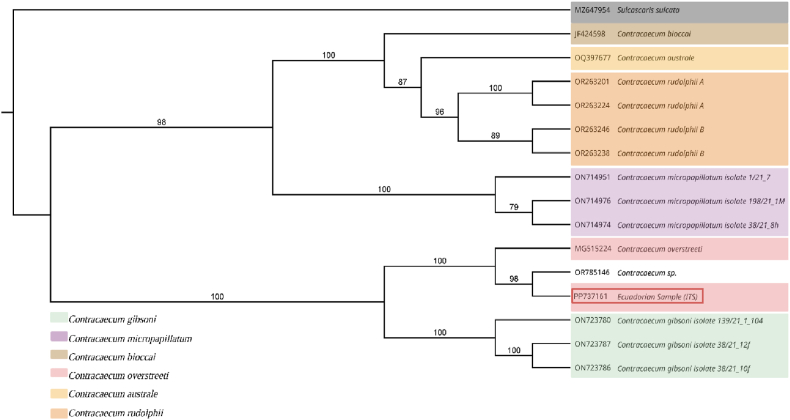
Neighbour-joining tree based on ITS sequences. Bootstrap support values (≥50 %) are shown at the nodes. The Ecuadorian sample (PP737161) highlighted in red rectangle and positioned within the *Contracaecum* spp. Clade. *Sulcascaris sulcata* was used as an outgroup.

BLAST analysis of the ITS1–5.8 S–ITS2 sequence revealed the highest similarity with members of the genus *Contracaecum*, particularly sequences assigned to *the C. overstreeti* clade. The phylogenetic tree inferred from ITS1–5.8 S–ITS2 sequences showed a well-defined structure within the genus *Contracaecum*, with bootstrap support values ranging from 79 to 100. The Ecuadorian specimen (PP737161) clustered with a previously deposited *Contracaecum* sp. sequence (OR785146), forming a robust clade (bootstrap = 98), which was resolved as a sister group to *C. overstreeti* (MG515224), supported by a bootstrap value of 100 (Fig. 3).

**Fig. 4:**
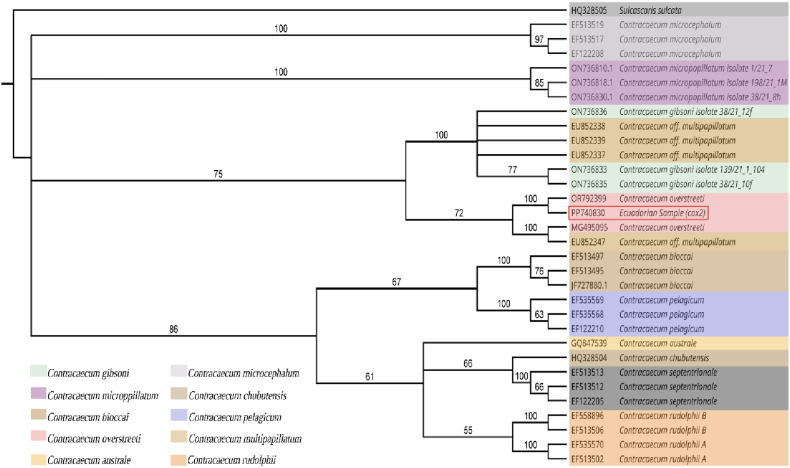
Neighbour-joining tree based on *cox2* sequences. Bootstrap support values (≥50 %) are shown at the nodes. The Ecuadorian sample (PP740830), marked within a red rectangle, is positioned within the *Contracaecum overstreeti* clade. *Sulcascaris sulcata* was used as an outgroup.

BLAST analysis of the mitochondrial *cox2* sequence showed its highest identity (99.6 %) with a sequence labelled as *C. overstreeti* (OR792399), corresponding to an unpublished GenBank record. In contrast, the *cox2* sequence showed a lower identity (92.64 %) with *Contracaecum* aff. *Multipapillatum* B (GenBank: EU852343), which represents the reference sequence of *C. overstreeti* according to its original species description. Phylogenetic analysis based on the *cox2* gene revealed a well-resolved topology within *Contracaecum*. The Ecuadorian sample (PP740830) grouped with sequences assigned to *C. overstreeti* (MG495095), supported by a bootstrap value of 100, and further clustered with *C. overstreeti* and *C*. aff. *Multipapillatum* sequences (OR792399 and EU852347) with strong support (bootstrap = 100) (Fig. 4).

Although minimal nucleotide divergence was observed (≤10 variable sites), both molecular markers consistently clustered within the *C. overstreeti* clade, as evidenced by high bootstrap support (≥98) in independent phylogenetic analyses.

## 4 Discussion

The present study provides the first molecular identification of the Anisakidae *Contracaecum overstreeti* larvae infecting the grey mullet (*Mugil cephalus*) fish in the northwestern Pacific coast of Ecuador. This finding expands the known diversity of anisakid nematodes in the country and highlights mugilids as previously unrecognized hosts in this region. Earlier research conducted in central and southern coastal areas of Ecuador reported *Anisakis pegreffii* and *A. physeteris* in several pelagic fish species, including bullet tuna (*Auxis rochei*), common dolphinfish (*Coryphaena hippurus*), skipjack tuna (*Katsuwonus pelamis*), and hake (*Merluccius gayi*) (Castellanos et al., 2018, see Fig. 1). That study using multiplex PCR with universal and species-specific primers, among them one targeting *Contracaecum osculatum* was unable to identify *Contracaecum species*, and importantly, did not include mugilids in its sampling analysis. Furthermore, the authors found the larvae in the viscera and mesenterium but not in the muscle tissue where we found the larvae, but not in the abdominal cavity and viscera. It could be an interesting difference in *Anisakis* and *Contracaecum* groups. The localization of anisakid larvae in fish muscle tissue, underscores higher risk of human infection through the consumption of raw or undercooked fish in traditional dishes. A human case of anisakidosis caused by *Contracaecum* sp. experiencing abdominal pain, vomiting and diarrhoea was reported in Australia after eating locally caught fish (Shamsi and Butcher, 2011).

Ecuadorian towns and cities host open marketplaces where various edible fishes, caught by artisanal techniques, are sold. Although fish are kept on ice, they are not refrigerated, and some vendors transport them in ice coolers to nearby communities. As fish are marketed without sanitary inspection, potential parasites remain unchecked. Vendors and consumers have observed worms in the muscle of the fish but are unaware of the parasite's identity or infection risks. It emphasizes the need for robust food safety measures, including thorough inspections, proper freezing techniques, and public education about the risks of consuming raw or undercooked fish (Rodríguez-Hidalgo et al., 2024). The high prevalence of fish infection encountered here is in accordance with Bracho (Bracho-Espinoza, 2023) who reported in Venezuela *Contracaecum* spp. parasitizes 100 % and 94 % of the fish of the families *Mugilidae* and *Gerreidae*, respectively. From Colombia are reports that the genera *Anisakis*, *Pseudoterranova*, and *Contracaecum* parasitized consumable fishes including *Mugil curema* and *M. cephalus* (Castellanos et al., 2017, 2018).

The molecular identification based on nuclear (ITS1-5.8 S-ITS2) and mitochondrial (*cox*2) markers provides compelling evidence for classifying the Ecuadorian larvae within the genus *Contracaecum*. The absence of amplification using *Anisakis simplex*–specific primers targeting elongation factor EF-1α further excludes their assignment to the genus *Anisakis*, reinforcing this taxonomic delimitation.

The mitochondrial *cox2* marker offered additional resolution regarding intra- and interspecific relationships within *Contracaecum*. The Ecuadorian sequence clustered with GenBank records assigned to *C. overstreeti* (MG495095 and OR792399) with maximal bootstrap support, indicating a high degree of mitochondrial similarity among geographically distant isolates. However, it is important to note that GenBank annotations within the *C. multipapillatum* species complex are not always taxonomically consistent. In particular, the mitochondrial sequence EU852343, deposited as *C. aff. Multipapillatum* B, represents the reference sequence of *C. overstreeti* according to its original species description based on integrated morphological and molecular evidence. (Mattiucci et al., 2010).

This discrepancy highlights the limitations of relying solely on BLAST-based identifications when dealing with species complexes characterized by historical mislabelling or provisional nomenclature. Accordingly, phylogenetic placement and reference to authoritative taxonomic frameworks are essential for accurate species-level interpretation within *Contracaecum*.

In contrast, the nuclear ITS marker placed the Ecuadorian larvae in a clade with a previously reported *Contracaecum* sp. sequence from Peru (OR785146), recovered as a sister group to *C. overstreeti*. This pattern is consistent with the limited availability of ITS1–5.8 S–ITS2 sequences for *Contracaecum species* in public databases and reflects the lower resolving power of this marker within closely related taxa of the *C. multipapillatum* complex.

The high fish infection rate encountered in this study may also have a negative impact on the mugilids health (FAO, 2016). *M. cephalus* has high capture rates by artisanal fishing, is an important source of proteins for Ecuadorians, and it is both fished and farmed. In Ecuador “lisa” is prepared raw in “ceviche” and salted and dried in “fanesca” dishes, increasing the chance of infection in consumers. Anisakidae larvae, even dead, could develop allergic reactions in some individuals (Audicana and Kennedy, 2008).

A limitation of the present study was the relatively small numbers of fish analysed. However, the high fish infection observed provides valuable preliminary insights and the fish were bought opportunistically from an open market. Future studies will focus on examining a larger number of specimens obtained systematically across different seasons and marketplaces to better understand the infection dynamics and prevalence.

## 5 Conclusions

Our finding confirms the presence of Anisakidae larvae in “lisa” fish of human consumption along the Ecuadorian Pacific coast, underscoring the need for further investigation into its possible emerging disease in Ecuador. To date, no human cases have been reported in the country, though dozens have been documented in neighbouring countries, including Peru, Colombia, Venezuela, and Chile (Falla-Zuñiga et al., 2021). Underdiagnosis may be likely, as the presence of anisakids in edible fish in Ecuador was only recognized in recent years (Castellanos et al., 2018) and corroborated by this study. It is expected that future research will reveal new species in fishes not yet examined and expand the host range of known species of Anisakidae.

## CRediT authorship contribution statement

**Manuel Calvopina:** Writing – review & editing, Writing – original draft, Validation, Supervision, Methodology, Investigation, Conceptualization. **Carlos Bastidas-Caldes:** Writing – review & editing, Validation, Methodology, Data curation. **Fernanda Hernández-Alomía:** Writing – review & editing, Methodology, Data curation. **William Cevallos:** Writing – review & editing. **Richar Rodríguez-Hidalgo:** Conceptualization. **Hiromu Sugiyama:** Writing – review & editing.

## Ethics approval and consent to participate

Not applicable. Ethical review and approval were not required for the present study because the investigations were performed on a dead commercial fish. No live animals were involved.

## Availability of data and materials

The sequences generated in the present study were submitted to the GenBank database under the accession numbers PP737161 (ITS2) and PP740830 (mtDNA *cox2*).

## Funding

This study was supported by the grant of Universidad de las Americas (UDLA) Quito (Grant No.490.B.XIV.24 to MC).

## Declarations of competing interests

The authors declare no competing interests.
